# Effect of the simulated half leaf width of a multileaf collimator on volumetric modulated arc therapy plan quality in hippocampal avoidance whole‐brain radiotherapy

**DOI:** 10.1002/acm2.13575

**Published:** 2022-03-03

**Authors:** Ming‐Hsien Li, Li‐Jhen Chen, Chih‐Chieh Chang, Jo‐Ting Tsai, Jang‐Chun Lin

**Affiliations:** ^1^ Department of Radiation Oncology Shuang Ho Hospital Taipei Medical University Taipei Taiwan ROC; ^2^ Department of Radiology School of Medicine College of Medicine Taipei Medical University Taipei Taiwan ROC

**Keywords:** hippocampus, leaf width, volumetric modulated arc therapy

## Abstract

**Purpose:**

Whole‐brain radiotherapy (WBRT) is commonly used in patients with multiple brain metastases. Compared with conventional WBRT, hippocampal avoidance WBRT (HA‐WBRT) more favorably preserves cognitive function and the quality of life. The hippocampal volume is considerably small (approximately 3.3 cm^3^). Therefore, downsizing the leaf width of a multileaf collimator (MLC) may provide higher spatial resolution and better plan quality. Volumetric modulated arc therapy (VMAT) could simulate the half MLC leaf width through couch shifting between arcs. This study investigated changes in VMAT quality for HA‐WBRT with a simulated fine MLC leaf width.

**Methods:**

We included 18 patients with brain metastasis. All target and avoidance structures were contoured by an experienced radiation oncologist. The prescribed dose was 30 Gy in 10 fractions. For each patient, three different treatment plans were generated for comparison: VMAT with couch‐shift, VMAT without couch‐shift, and TomoTherapy. All treatment plans fulfilled Radiation Therapy Oncology Group (RTOG) 0933 criteria for HA‐WBRT. The Wilcoxon paired signed‐rank test was used to compare different treatment plans.

**Results:**

VMAT with couch‐shift had the better average conformity index (0.823) with statistically significant difference compared to VMAT without couch‐shift (0.810). VMAT with couch‐shift (0.219) had a more favorable average homogeneity index (HI) than did VMAT without couch‐shift (0.230), although the difference was not significant. TomoTherapy had an optimal average HI of 0.070. In terms of the hippocampus, all three treatment plans met the RTOG 0933 criteria. VMAT with couch‐shift had a lower average *D*_max_ (15.2 Gy) than did VMAT without couch‐shift (15.3 Gy, *p* = 0.071) and TomoTherapy (15.5 Gy, *p* = 0.133). The average *D*_100%_ of hippocampus was the same for both VMAT with and without couch‐shift (8.5 Gy); however, TomoTherapy had a lower average *D*_100%_ value of 7.9 Gy. The treatment delivery time was similar between VMAT with and without couch‐shift (average, 375.0 and 369.6 s, respectively). TomoTherapy required a long average delivery time of 1489.9 s.

**Conclusion:**

The plan quality of VMAT for HA‐WBRT was improved by using the couch‐shift technique to simulate the half MLC leaf width. However, the improvement was not statistically significant except conformity index. The downsizing effect decreased with the use of the sophisticated grade of VMAT. TomoTherapy offered superior plan quality but required the longest delivery time.

## 1 INTRODUCTION

Brain metastasis occurs in 10–30% of patients with cancer. As effective systemic treatment regimens have been demonstrated to prolong life, the incidence of brain metastasis is increasing. Whole‐brain radiotherapy (WBRT) is most commonly used in patients with numerous brain metastases because it can alleviate symptoms and substantially improve intracranial control. Approximately 40–60% of patients respond to WBRT.^1^, ^2^ However, WBRT is associated with many neurological side effects including the development of dementia, cerebellar dysfunction, and deficits in neurocognitive functions, such as short‐term memory.

Deficits in learning, memory, and spatial processing occurring in patients undergoing WBRT are believed to be related to hippocampal injury.^3^ Several studies have demonstrated that cognitive function is associated with radiosensitive neural stem cells within the hippocampal dentate gyrus.^4^ Radiation Therapy Oncology Group (RTOG) 0933 is a multi‐institution phase II trial investigating the effectiveness of hippocampal avoidance (HA) during WBRT for brain metastasis. The trial revealed that HA‐WBRT exhibited a stronger association with the preservation of memory and quality of life in patients with brain metastasis than in historical controls.^5^

Because the hippocampus is located in the deep central area of the brain, its optimization is considerably difficult because the main organ at risk is completely surrounded by the planning target volume (PTV). Therefore, RTOG 0933 suggested using intensity‐modulated radiotherapy (IMRT), helical TomoTherapy, or volumetric arc therapy (VMAT). In addition, the hippocampal volume is substantially small; on average, it is 3.3 cm^3^ and accounts for 2.1% of the whole‐brain PTV.^6^ Downsizing the leaf width of MLC may provide higher spatial resolution and better radiotherapy plan quality. Abisheva et el. investigated the effect of the leaf width of an MLC on single‐isocenter multitarget radiosurgery with VMAT and found that a 2.5‐mm‐wide MLC had a lower isodose spill than did a 5‐mm‐wide MLC.^7^ In addition, VMAT could simulate the dosimetric effect of the half MLC leaf width with couch shift between arcs.^8^

In this study, we investigated changes in the VMAT quality for HA‐WBRT with a simulated fine MLC leaf width. By using an MLC with a 1‐cm‐wide leaf on an Elekta Synergy linear accelerator, we simulated 5‐mm‐wide leaves with a 5‐mm shift of the patient couch in the longitudinal direction between arcs.

## 2 METHODS

### 2.1 Patient selection, delineation, and planning constraints

This study was approved by the institutional review board of our institution. A total of 18 patients with brain metastasis treated with HA‐WBRT were reviewed. Patients underwent a noncontrast computed tomography (CT) simulation scan with a slice thickness of 3 mm. The CT simulation and MRI were fused, and target and avoidance structures were contoured by an experienced radiation oncologist by using the Pinnacle^3^ version 14 planning software. The hippocampus was contoured on T1‐weighted MRI axial sequences in accordance with RTOG 0933 contouring guidelines. The HA region was generated through a 5‐mm three‐dimensional expansion of the hippocampal contour. The clinical target volume (CTV) was defined as the whole‐brain parenchyma to C1, and the PTV was defined as the CTV excluding the HA region. Other normal structures that were contoured included the optic nerves, optic chiasm, lenses, and inner ears. The inner ears included the cochlea and internal auditory meatus. Following RTOG guidelines, the treatment prescribed was a delivery of 30 Gy in 10 fractions to the PTV. The RTOG 0933 acceptable compliance criteria for target and normal tissue planning doses are as follows:

1. At least 90% of the brain volume should receive 30 Gy (*V*_30Gy_ ≥ 90% PTV).
2. 2% of the target volume should receive 37.5 Gy or less (*D*_2%_ ≤ 37.5 Gy).
3. 98% of the target volume should receive 25 Gy or more (*D*_98%_ ≥ 25 Gy).
4. The minimum dose to the hippocampus (*D*_min_ = *D*_100%_) should be ≤9 Gy.
5. The maximum dose to the hippocampus should be ≤16 Gy.
6. The maximum dose to the optic nerves and chiasm should be ≤37.5 Gy.

### 2.2 VMAT planning

VMAT plans were generated using 6‐MV photon beams and optimized using Pinnacle^3^ Version 14 on the Elekta Synergy platform with 40 MLC leaf pairs with a leaf width of 1 cm at the isocenter. For each patient, two VMAT plans were generated: one without couch‐shift and one with couch‐shift in the longitudinal direction between full arcs (FAs). The VMAT plan without couch‐shift was generated using four FAs (clockwise for two FAs and counterclockwise for two FAs) without couch‐shift between arcs. The collimator angle was 45°. The VMAT plan with couch‐shift was also generated using the same four FAs as the VMAT plan without couch‐shift. But we shifted the patient couch by 5 mm in the longitudinal direction after the two FAs (one clockwise FA and one counterclockwise FA). The collimator angle in this plan was also 45°.

### 2.3 TomoTherapy planning

TomoTherapy plans were generated using the Hi‐ART planning system (TomoTherapy Inc, Version 5.1.4, Madison, WI, USA). The plan parameters were as follows: field width, 1.05 cm; pitch, 0.215; and modulation factor, 3.0. The plans were optimized such that 96% of the whole‐brain PTV received the prescription dose of 30 Gy in 10 fractions.

### 2.4 Plan evaluation

For the PTV, the volume receiving > 30 Gy (*V*_30Gy_) and the minimum dose covering 98% of the volume (*D*_98%_) were used to assess coverage. The dose delivered to the hottest 2% of the PTV (*D*_2%_) was used to determine the number of hotspots in the treatment plan. The conformity index (CI) and homogeneity index (HI) were defined as follows:

(1) $$\mathrm{HI}=\left(D_{2\%}-D_{98\%}\right)/D_{50\%}$$

(2) $$\mathrm{CI}=\left(TV_{\mathrm{RI}}\times TV_{\mathrm{RI}}\right)/\left(TV\times V_{\mathrm{RI}}\right)$$

where *V*_RI_ is the total volume in the body receiving the prescribed dose, *TV* is the volume of PTV, and *TV*_RI_ is the volume of TV within *V*_RI_. Smaller HI values closer to 0 indicate superior homogeneity, whereas larger values closer to 1 indicate inferior homogeneity. The ideal and maximum CI value is 1, with a larger CI indicating better conformality.

For the hippocampus and HA ring, the maximum, minimum, and mean doses were extracted for comparison. Other normal tissue doses extracted for comparison in this study included the minimum and maximum doses to the optic nerves, optic chiasm, lenses, and inner ears.

In addition, the delivery time for a single fraction of HA‐WBRT was recorded for all three modalities and did not include the time taken for pretreatment patient setup and daily imaging procedures.

### 2.5 Statistical analysis

Statistical comparisons of the treatment plans between the three modalities were performed using a Wilcoxon paired signed‐rank test with the SPSS Version 25 statistical software (IBM, USA). A *p* value of ≤0.05 was considered statistically significant.

## 3 RESULTS

**FIGURE 1:**
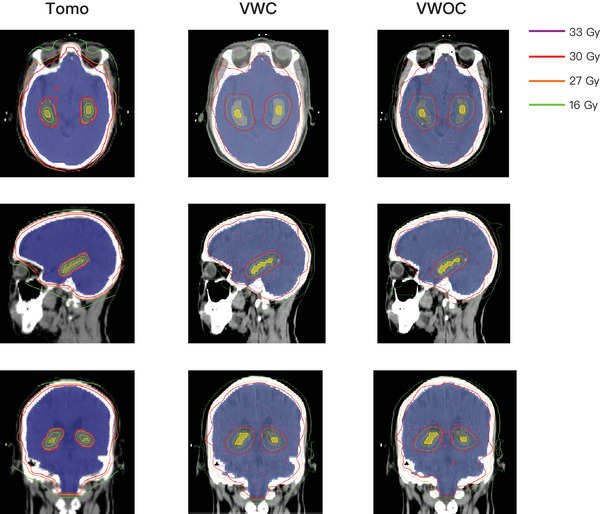
The dose distribution for one sample patient using the three treatments. *Note*: Tomo: TomoTherapy; VWC: VMAT with couch‐shift; VWOC: VMAT without couch‐shift; blue area: PTV; yellow area: hippocampus

Table 1 lists the characteristics of the 18 patients. Table 2 presents the average dosimetric values for the 18 patients. The results of the comparison among TomoTherapy, VMAT without couch‐shift, and VMAT with couch‐shift performed using the Wilcoxon paired signed‐rank test are shown in Table 2, and *p* values are summarized in Table 3. Figure 1 displays the dose distribution from the three treatment modalities for one sample patient in our study. Average accumulated dose‐volume histogram (DVH) of the PTV and the hippocampus is shown in Figure 2. The treatment plans for all the patients were in compliance with RTOG 0933 protocol dosimetric criteria.

**TABLE 1:** Patient characteristics and volumes for PTV and hippocampus

Patient no.	Primary tumor	PTV (ml)	Hippocampus (ml)
1	Breast cancer	1226.02	2.72
2	Breast cancer	1286.74	2.83
3	Cervical cancer	1236.50	2.41
4	Liver cancer	1378.30	3.21
5	Lung cancer	1248.99	3.41
6	Lung cancer	1277.47	3.30
7	Lung cancer	1449.23	3.16
8	Lung cancer	1364.34	2.33
9	Lung cancer	1257.80	2.49
10	Lung cancer	1287.56	2.37
11	Lung cancer	1086.26	3.03
12	Lung cancer	1317.77	2.97
13	Lung cancer	1251.98	2.65
14	Lung cancer	1394.67	2.73
15	Lung cancer	1373.62	2.96
16	Lung cancer	1098.94	2.71
17	Lung cancer	1485.25	3.06
18	Lung cancer	1142.99	3.01
Average volume		1286.91	2.85

**TABLE 2:** Average dosimetric values and comparison of the three treatments (*n* = 18)

Structure	Dosimetry metric (protocol criteria)	TomoTherapy	VWC	VWOC	Tomo vs. VWC	Tomo vs. VWOC	VWC vs. VWOC
PTV	*V*_30Gy_ (≥90%)	96.56%	92.95%	92.90%	**	**	NS
	*D*_2%_ (≤37.5 Gy)	31.7 Gy	33.0 Gy	33.2 Gy	**	**	NS
	*D*_98%_ (≥25 Gy)	29.5 Gy	26.1 Gy	25.9 Gy	**	**	NS
	HI	0.070	0.219	0.230	**	**	NS
	CI	0.815	0.823	0.810	NS	NS	*
PIV (ml)		1477.11	1351.11	1372.27			
Hippocampus	*D*_max_	15.5 Gy	15.2 Gy	15.3 Gy	NS	NS	NS
	*D*_min_	7.9 Gy	8.5 Gy	8.5 Gy	**	**	NS
	*D*_mean_	10.7 Gy	11.2 Gy	11.4 Gy	*	**	NS
HA ring	*D*_max_	29.7 Gy	25.6 Gy	25.7 Gy	**	**	NS
	*D*_min_	9.2 Gy	8.7 Gy	8.7 Gy	NS	NS	NS
	*D*_mean_	18.7 Gy	16.3 Gy	16.3 Gy	**	**	NS
Left optic nerves	*D*_max_	30.0 Gy	31.7 Gy	32.0 Gy	**	**	NS
	*D*_mean_	26.4 Gy	25.4 Gy	25.3 Gy	*	*	NS
Right optic nerves	*D*_max_	30.0 Gy	31.7 Gy	32.2 Gy	**	**	NS
	*D*_mean_	26.0 Gy	24.6 Gy	25.4 Gy	**	NS	NS
Left lenses	*D*_max_	4.2 Gy	4.4 Gy	4.7 Gy	NS	*	NS
	*D*_mean_	3.2 Gy	3.9 Gy	4.1 Gy	**	**	*
Right lenses	*D*_max_	4.2 Gy	4.4 Gy	4.6 Gy	NS	*	NS
	*D*_mean_	3.2 Gy	3.8 Gy	4.0 Gy	**	**	NS
Left inner ear	*D*_max_	30.6 Gy	32.2 Gy	32.7 Gy	**	**	NS
	*D*_mean_	28.3 Gy	30.6 Gy	30.8 Gy	**	**	NS
Right inner ear	*D*_max_	31.0 Gy	32.3 Gy	32.2 Gy	**	*	NS
	*D*_mean_	28.5 Gy	30.4 Gy	30.6 Gy	*	*	NS
Optic chiasm	*D*_max_	31.8 Gy	33.9 Gy	33.8 Gy	**	**	NS
MUs		21503.6	1141.1	1099.8	**	**	NS
Delivery time		1489.9 s	375.0 s	369.6 s	**	**	NS

Abbreviations: CI, conformity index; HA, hippocampal avoidance; HI, homogeneity index; NS, not significant; PIV, prescription isodose volume; VWC, VMAT with couch‐shift; VWOC, VMAT without couch‐shift.

* *p*≤0.05; **p≤0.005.

**TABLE 3:** The *p*‐value list of PTV dosimetric analysis (*n* = 18)

Structure	Dosimetry metric	Tomo vs. VWC	Tomo vs. VWOC	VWC vs. VWOC
PTV	*V*_30Gy_	0.000	0.000	0.965
	*D*_2%_	0.000	0.000	0.122
	*D*_98%_	0.000	0.000	0.463
	HI	0.000	0.000	0.199
	CI	0.616	0.557	0.039
Hippocampus	*D*_max_	0.133	0.420	0.071
	*D*_min_	0.003	0.002	0.777
	*D*_mean_	0.008	0.004	0.396
HA ring	*D*_max_	0.000	0.000	0.500
	*D*_min_	0.157	0.112	0.845
	*D*_mean_	0.000	0.000	0.948
Left optic nerves	*D*_max_	0.001	0.001	0.500
	*D*_mean_	0.012	0.012	0.913
Right optic nerves	*D*_max_	0.005	0.001	0.133
	*D*_mean_	0.001	0.170	0.071
Left lenses	*D*_max_	0.231	0.028	0.112
	*D*_mean_	0.000	0.000	0.016
Right lenses	*D*_max_	0.122	0.008	0.286
	*D*_mean_	0.000	0.000	0.133
Left inner ear	*D*_max_	0.000	0.000	0.145
	*D*_mean_	0.000	0.001	0.528
Right inner ear	*D*_max_	0.005	0.014	0.879
	*D*_mean_	0.006	0.007	0.215
Optic chiasm	*D*_max_	0.000	0.000	0.744
MUs		0.000	0.000	0.133
Delivery time		0.000	0.000	0.632

Abbreviation: CI , conformity index; HA, hippocampal avoidance; HI, homogeneity index; VWC, VMAT with couch‐shift; VWOC, VMAT without couch‐shift.

FIGURE 2Average dose–volume histogram of the PTV

**Figure:**
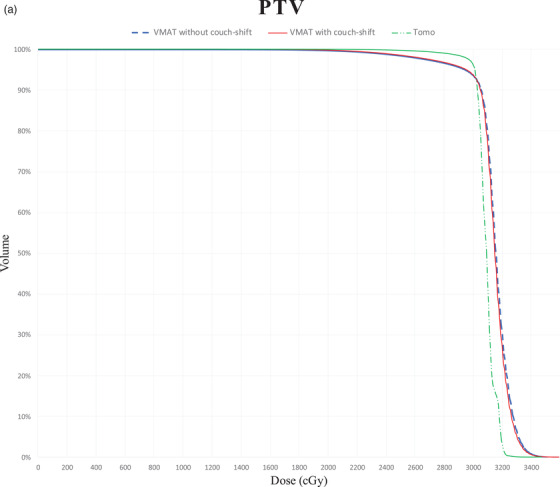

**Figure:**
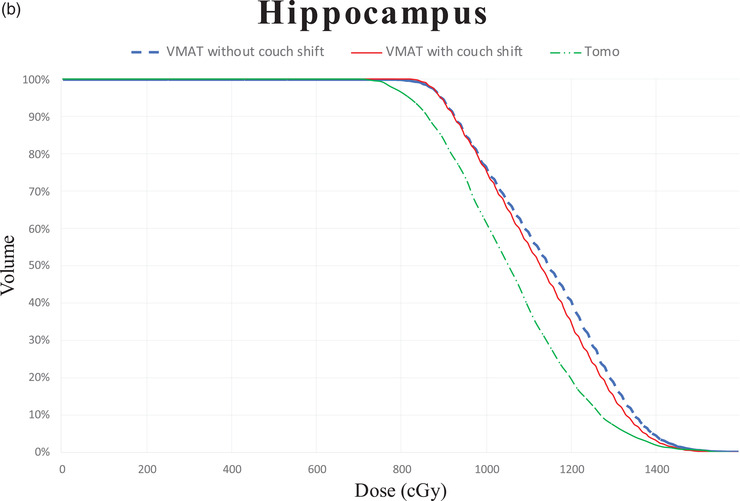

### 3.1 PTV

TomoTherapy provided the most satisfactory PTV coverage among the modalities, with an average *V*_30Gy_ of 96.56%. The VMAT plans with and without couch‐shift had similar average *V*_30Gy_ values of 92.95% and 92.90%, respectively. The average CI of the three treatment plans was 0.815 for TomoTherapy, 0.823 for VMAT with couch‐shift, and 0.810 for VMAT without couch‐shift. The VMAT plan with couch‐shift had the optimal CI. It may be that the average prescription isodose volume in TomoTherapy was 1477.11 ml, which was larger than the average prescription isodose volume in the VMAT plans. Regarding hotspots, the average *D*_2%_ for TomoTherapy (31.7 Gy) was significantly lower than that for the VMAT plans. Moreover, the average *D*_2%_ values did not significantly differ between the VMAT plans with and without couch‐shift (33.0 and 33.2 Gy, respectively). Regarding the minimum target dose criteria *D*_98%_, TomoTherapy (29.5 Gy) exhibited a significantly higher value than did VMAT with couch‐shift (26.1 Gy) and VMAT without couch‐shift (25.9 Gy). Furthermore, TomoTherapy had an average HI of 0.070, which was more favorable than that for VMAT with couch‐shift (0.219) and VMAT without couch‐shift (0.230).

### 3.2 Hippocampus and HA ring

In terms of the dose to the hippocampus, both VMAT with (8.5 Gy) and without (8.5 Gy) couch‐shift had a higher average *D*_100%_ than did TomoTherapy (7.9 Gy). However, all the doses were within the protocol's recommendation of 9 Gy. In terms of *D*_100%_, TomoTherapy was significantly superior to VMAT with and without couch‐shift. No difference in *D*_100%_ was noted between VMAT with and without couch‐shift. VMAT with couch‐shift had a lower average *D*_max_ (15.2 Gy) than did VMAT without couch‐shift (15.3 Gy, *p* = 0.071) and TomoTherapy (15.5 Gy, *p* = 0.133). In terms of the average *D*_mean_ of the hippocampus, TomoTherapy (10.7 Gy) had significantly lower values than did VMAT with (11.2 Gy) and without (11.4 Gy) couch‐shift.

In terms of HA ring, TomoTherapy (18.7 Gy) had a higher average *D*_mean_ than did VMAT with (16.3 Gy) and without (16.3 Gy) couch‐shift. No significant difference in the average *D*_mean_ was observed between VMAT with and without couch‐shift.

### 3.3 Optic nerves and optic chiasm

For the optic nerves and chiasm, VMAT with and without couch‐shift as well as TomoTherapy met the RTOG 0933 constraint (*D*_max_ ≤ 37.5 Gy). TomoTherapy had a lower average *D*_max_ value than did both VMAT with and without couch‐shift. No significant difference in *D*_max_ values was observed between VMAT with and without couch‐shift. TomoTherapy had a higher average *D*_mean_ value for the optic nerves than did VMAT with and without couch‐shift. The average *D*_mean_ value of the optic nerves was similar for VMAT with and without couch‐shift.

### 3.4 Lenses and inner ear

RTOG 0933 did not set any dose constraints for the lens and inner ear. In this study, TomoTherapy had lower average values of the mean and maximal doses for the lens (*D*_max_ = 4.2 Gy; *D*_mean_ = 3.2 Gy) and inner ear (*D*_max_ = 31.0 Gy; *D*_mean_ = 28.4 Gy) than VMAT with and without couch‐shift. For the inner ear, VMAT with couch‐shift had average *D*_max_ and average *D*_mean_ (32.3 and 30.5 Gy, respectively) comparable to those of VMAT without couch‐shift (*D*_max_ = 32.7 Gy; *D*_mean_ = 30.7 Gy).

### 3.5 Delivery time

TomoTherapy required the longest treatment delivery time (1489.9 s) averaging over 18 patients. VMAT with couch‐shift had an average delivery time of 375.0 s, which was similar to that of VMAT without couch‐shift (369.6 sec). TomoTherapy had almost four times longer delivery time than did VMAT with and without couch‐shift. Notably, the delivery time was an estimated value recorded from the time the first beam delivery was started to the end of the last beam delivery.

## 4 DISCUSSION

When all the three modalities met RTOG 0933′s dosimetric criteria, we found that VMAT with couch‐shift yielded a better CI (average CI = 0.823) and TomoTherapy yielded a better HI (average HI = 0.070). TomoTherapy provided the optimal PTV coverage (*V*_30Gy_ = 96.56%); however, the average prescription isodose volume in TomoTherapy was considerably larger than the average PTV (prescription isodose volume = 1477.11 mL; PTV = 1286.91 mL). Thus, VMAT with couch‐shift (0.823) yielded a better CI than did TomoTherapy (0.815), but the difference did not have statistical significance. VMAT with couch‐shift could simulate the half width of an MLC. In our study, we observed a significant difference in CI between VMAT with and without couch‐shift. With respect to the dose to the hippocampus, VMAT with couch‐shift had a lower average *D*_max_ (15.2 Gy) than did VMAT without couch‐shift (15.3 Gy, *p* = 0.071).

In 2016, Park et al. investigated the effect of an extremely narrow‐width MLC leaf on the plan quality of VMAT for prostate cancer.^8^ They simulated 2.5‐mm‐wide MLCs by shifting the isocenter position by 2.5 mm with 5‐mm‐wide MLCs. The profile of the 2.5‐mm MLC simulation was almost the same as that of the real 2.5‐mm wide MLCs. The Halcyon IMRT/VMAT delivery system is composed of two staggered layers of 28 leaf pairs, with a projected leaf width of 10 mm. This double‐staggered MLC has an effective leaf resolution of 5 mm. Several studies have compared the plan quality between a Halcyon and a TrueBeam, which comprises 40 central leaf pairs with a width of 5 mm and 20 peripheral leaf pairs with a width of 10 mm.^9^, ^10^ These studies have found that the Halcyon could provide a plan quality similar to that of the TrueBeam for VMAT techniques. In the present study, we used the VMAT plan with couch‐shift in the longitudinal direction between arcs to simulate a fine MLC leaf width.

The leaf width of MLCs is crucial for target shaping resolution. A thin leaf width generally results in higher resolution and therefore provides more favorable dosimetric outcomes in IMRT planning. Several studies have investigated the dosimetric benefits of thin‐leaf MLCs in IMRT and have shown that thin‐leaf MLCs in IMRT yielded better target coverage, CI, and HI.^11^, ^12^, ^13^ However, the downsizing effect of the MLC leaf width decreased with the use of the sophisticated grades of IMRT and VMAT.^14^ With respect to normal tissue sparing, dosimetric improvements still decreased with the use of the sophisticated grades of techniques (IMRT or VMAT).^14^, ^15^ Chae et al. investigated the dosimetric effects of leaf width on IMRT and VMAT for small‐sized C‐shape and large‐sized head and neck phantoms and found that the thin leaf could yield higher CI and a lower spinal cord dose for C‐shape. In our study, we used a couch shift between arcs in VMAT to simulate the half width of MLCs for HA‐WBRT. The shape of PTV was similar to that of a doughnut. The thin leaf provided a higher average CI (from 0.810 to 0.823, *p* = 0.039) and a more favorable average HI (from 0.230 to 0.219, *p* = 0.199) for PTV. Moreover, the thin leaf could reduce the average maximum hippocampus dose (from 15.3 to 15.2 Gy, *p* = 0.071) and the average mean hippocampus dose (from 11.4 to 11.2 Gy, *p* = 0.396), although the results were not significant. The results of the present study are consistent with those of the aforementioned studies.

Pigorsch et al. compared the dosimetry of three different plans (VMAT, IMRT, and TomoTherapy) for head and neck cancer.^16^ The formula of CI was the same as ours. They used the technique of simultaneous integrated boost (SIB). Best CIs for SIB77Gy and SIB70Gy were achieved by TomoTherapy and the best CI for SIB56Gy was achieved by VMAT. The best performance for HI in all SIBs was achieved only by TomoTherapy. In our study, VMAT with couch‐shift provided better CI (0.823) than did TomoTherapy (0.815), although the difference was not statistically significant. TomoTherapy provided the optimal HI. DVH also showed that TomoTherapy had steepest dose gradient of PTV.

In clinical practice, the prerequisite of the VMAT with couch‐shift technique is an accurate setup of a patient based on 3D image guidance. In this study, we used an Elekta Synergy linear accelerator. And we moved the patient couch 5 mm in the longitudinal direction between arcs to simulate half leaf width. In a previous study, the positioning error of Elekta Synergy was 0.11 ± 0.12 mm in the longitudinal direction.^17^ This technique should be applied with couches that could operate with submillimeter precision.^17^, ^18^

In the present study, TomoTherapy yielded the optimal plan quality but required the longest delivery time. The plan parameters were in accordance with the planning recommendations of RTOG 0933 (field width, 1.05 cm; pitch, 0.215; and modulation factor, 3.0). The plan quality and treatment time depend on the field width, rotation pitch, and modulation factor. De kerf et al. investigated the optimal parameters for TomoTherapy and found that the treatment time was reduced by almost 50% when they used a field width of 5 cm compared with a field width of 2.5 cm.^19^ However, the dose distribution was not cost‐effective. Furthermore, Van Gestel et al. attempted to reduce TomoTherapy treatment time by modifying the field width from 2.5 to 5.0 cm, increasing the pitch, and lowering the modulation factor.^20^ Although the plan quality deteriorated, it was still acceptable. Further investigation is warranted to identify the optimal parameters to obtain high delivery efficiency as well as quality when using TomoTherapy for HA‐WBRT.

## 5 CONCLUSION

Patients with multiple brain metastases may not tolerate the prolonged radiation treatment time. VMAT can facilitate efficient HA‐WBRT while still meeting the RTOG 0933 dosimetric criteria. By shifting the couch in the longitudinal direction between arcs, the VMAT plan quality could be improved. TomoTherapy offered superior plan quality but required the longest delivery time for HA‐WBRT. Thus, TomoTherapy may be not suitable for patients with multiple brain metastases. Additional studies are warranted to identify the optimal parameters for achieving high efficiency and quality in TomoTherapy.

## CONFLICT OF INTEREST

The authors declare no conflicts of interest.

## AUTHOR CONTRIBUTION STATEMENT

Ming‐Hsien Li conceived and planned the experiment. Ming‐Hsien Li, Li‐Jhen Chen and Chih‐Chieh Chang carried out the experiment. Ming‐Hsien Li wrote the manuscript with support from Jo‐Ting Tsai and Jang‐Chun Lin.

## References

[1] Mehta MP , Rodrigus P , Terhaard CH , et al. Survival and neurologic outcomes in a randomized trial of motexafin gadolinium and whole‐brain radiation therapy in brain metastases. J Clin Oncol. 2003;21(13):2529‐2536. 12829672 10.1200/JCO.2003.12.122

[2] Suh JH , Stea B , Nabid A , et al. Phase III study of efaproxiral as an adjunct to whole‐brain radiation therapy for brain metastases. J Clin Oncol. 2006;24(1):106‐114. 16314619 10.1200/JCO.2004.00.1768

[3] Abayomi OK . Pathogenesis of irradiation‐induced cognitive dysfunction. Acta Oncol. 1996;35(6):659‐663. 8938210 10.3109/02841869609083995

[4] Eriksson PS , Perfilieva E , Bjork‐Eriksson T , et al. Neurogenesis in the adult human hippocampus. Nat Med. 1998;4(11):1313‐1317. 9809557 10.1038/3305

[5] Gondi V , Pugh SL , Tome WA , et al. Preservation of memory with conformal avoidance of the hippocampal neural stem‐cell compartment during whole‐brain radiotherapy for brain metastases (RTOG 0933): a phase II multi‐institutional trial. J Clin Oncol. 2014;32(34):3810‐3816. 25349290 10.1200/JCO.2014.57.2909 PMC4239303

[6] Gondi V , Tolakanahalli R , Mehta MP , et al. Hippocampal‐sparing whole‐brain radiotherapy: a “how‐to” technique using helical tomotherapy and linear accelerator‐based intensity‐modulated radiotherapy. Int J Radiat Oncol Biol Phys. 2010;78(4):1244‐1252. 20598457 10.1016/j.ijrobp.2010.01.039 PMC2963699

[7] Abisheva Z , Floyd SR , Salama JK , et al. The effect of MLC leaf width in single‐isocenter multi‐target radiosurgery with volumetric modulated arc therapy. J Radiosurg SBRT. 2019;6(2):131‐138. 31641549 PMC6774495

[8] Park JM , Park SY , Kim JH , Carlson J , Kim JI . The effect of extremely narrow MLC leaf width on the plan quality of VMAT for prostate cancer. Radiat Oncol. 2016;11:85. 27338929 10.1186/s13014-016-0664-0 PMC4917980

[9] Michiels S , Poels K , Crijns W , et al. Volumetric modulated arc therapy of head‐and‐neck cancer on a fast‐rotating O‐ring linac: plan quality and delivery time comparison with a C‐arm linac. Radiother Oncol. 2018;128(3):479‐484. 29739713 10.1016/j.radonc.2018.04.021

[10] Sun T , Lin X , Zhang G , Qiu Q , Li C , Yin Y . Treatment planning comparison of volumetric modulated arc therapy with the trilogy and the Halcyon for bilateral breast cancer. Radiat Oncol. 2021;16(1):35. 33602267 10.1186/s13014-021-01763-z PMC7890882

[11] Wang S , Gong Y , Xu Q , et al. Impacts of multileaf collimators leaf width on intensity‐modulated radiotherapy planning for nasopharyngeal carcinoma: analysis of two commercial elekta devices. Med Dosim. 2011;36(2):153‐159. 20488691 10.1016/j.meddos.2010.02.007

[12] Hong C‐S , Ju SG , Kim M , et al. Dosimetric effects of multileaf collimator leaf width on intensity‐modulated radiotherapy for head and neck cancer. Med Phys. 2014;41:021712. 24506603 10.1118/1.4860155

[13] Zwicker F , Hauswald H , Nill S , et al. New multileaf collimator with a leaf width of 5 mm improves plan quality compared to 10 mm in step‐and‐shoot IMRT of HNC using integrated boost procedure. Strahlenther Onkol. 2010;186(6):334‐343. 20495969 10.1007/s00066-010-2103-8

[14] Chae SM , Lee KW , Son SH . Dosimetric impact of multileaf collimator leaf width according to sophisticated grade of technique in the IMRT and VMAT planning for pituitary adenoma lesion. Oncotarget. 2016;7(47):78119‐78126. 27806336 10.18632/oncotarget.12974 PMC5363648

[15] Amoush A , Long H , Subedi L , Qi P , Djemil T , Xia P . Dosimetric effect of multileaf collimator leaf width on volumetric modulated arc stereotactic radiotherapy for spine tumors. Med Dosim. 2017;42(2):111‐115. 28457723 10.1016/j.meddos.2017.01.007

[16] Pigorsch SU , Kampfer S , Oechsner M , et al. Report on planning comparison of VMAT, IMRT and helical tomotherapy for the ESCALOX‐trial pre‐study. Radiat Oncol. 2020;15(1):253. 33138837 10.1186/s13014-020-01693-2 PMC7607845

[17] Li W , Moseley DJ , Manfredi T , Jaffray DA . Accuracy of automatic couch corrections with on‐line volumetric imaging. J Appl Clin Med Phys. 2009;10(4):106‐116. 19918232 10.1120/jacmp.v10i4.3056 PMC5720567

[18] Riis HL , Zimmermann SJ . Elekta precise table characteristics of IGRT remote table positioning. Acta Oncol. 2009;48(2):267‐270. 18756401 10.1080/02841860802311007

[19] De Kerf G , Van Gestel D , Mommaerts L , Van den Weyngaert D , Verellen D . Evaluation of the optimal combinations of modulation factor and pitch for helical TomoTherapy plans made with TomoEdge using Pareto optimal fronts. Radiat Oncol. 2015;10(1):191. 26377574 10.1186/s13014-015-0497-2 PMC4573943

[20] Van Gestel D , De Kerf G , Wouters K , et al. Fast helical tomotherapy in a head and neck cancer planning study: is time priceless?. Radiat Oncol. 2015;10(1):261. 26701749 10.1186/s13014-015-0556-8 PMC4690403

